# Observations on Functional Disorders of the Spinal Cord, and Their Connexion with Hysterical, Nervous, and Other Diseases; Illustrated by Cases, Selected Chiefly from the Reports of the Pallas-Kenry and Currah Dispensaries

**Published:** 1830-05

**Authors:** Wm. Griffin, D. Griffin

**Affiliations:** Limerick.; Limerick.


THE LONDON
### Medical and Physical Journal.

No. 375, VOL. LXIII.] MAY 1830. [No. 47, *New Series.*

For many fortunate discoveries in medicine, and for the detection of numerous errors, the world is indebted to the rapid circulation of Monthly Journals; and there never existed any work to which the Faculty, in Europe and America, were under deeper obligations than to the *Medical and Physical Journal of London*, now forming a long but an invaluable series.—*Rush.*

### ORIGINAL PAPERS, AND CASES,
### OBTAINED FROM PUBLIC INSTITUTIONS AND OTHER AUTHENTIC SOURCES.

### FUNCTIONAL DISORDERS OF THE SPINAL CORD.

*Observations on Functional Disorders of the Spinal Cord, and their Connexion with Hysterical, Nervous, and other Diseases; illustrated by Cases, selected chiefly from the Reports of the Pallas-Kenry and Currah Dispensaries.* By WM. GRIFFIN, M.D. and D. GRIFFIN, M.R.C.S. Limerick.

(Continued from page 316.)

*Respiratory System* (*continued*).

IN our early practice we chanced to meet with many cases of inflammatory croup, and had just reason to acknowledge its fatality when not actively treated within the first twelve hours. With this experience, it surprised us not a little to hear respectable practitioners sometimes assert that they had few or no fatal cases, and that, at almost any period of the disease, they were able to effect cures, with the very remedies which with us were totally unavailing. In the course of time, however, instances of croup fell in our own way, which, though presenting few, and often no distinctive marks from the former, terminated favorably under the mildest treatment, and occasionally without any treatment at all. As it seemed very inconclusive reasoning to say these diseases differed only in degree, when they both presented symptoms equally violent, or when, in fact, that which was sure to end favorably was sometimes the more intense of the two, we at once concluded that, although attacking the same parts, &c., and therefore almost necessarily inducing the same symptoms, they differed altogether in their nature. Dr. Kellie agrees with his friend, Dr. Cheyne, in the opinion that, except in the circumstance of intermission, and continuance, and more or less violence of disease, there is not any real or essential difference: but is not, we may ask, regular intermission in itself a wide mark of distinction? Who would speak of intermittent and continued inflammation of any other organ of the body? Who would infer that hysteralgia and hysteritis, or spasm of the stomach and gastritis, which frequently differ only as to intermission and continuance, were but varieties of the same complaint? And surely, after all, we have the obvious fact before us, that in the very worst attacks of the one, even when neglected, patients most frequently recover; while in the mildest of the other, under the same circumstances, they almost invariably die. We wish we could offer post-mortem evidence of the different nature of these diseases, but that is exceedingly difficult to come at, and can only be obtained by the close observation and long experience of the profession at large. It seems, indeed, hardly just to demand proofs from appearances in the dead body, in diseases like those of irritation, which are so seldom fatal.

Dr. Cheyne says that, "until the advocates for the separate existence of spasmodic croup more fully assign and establish their grounds of belief, he is convinced it will be for the benefit of the patient that we should act as if there was but one kind." As the well-earned reputation of so eminent and experienced a practitioner necessarily gives great weight to any opinions he may offer, and as we feel the great importance of the distinction we have been contending for, as regards the treatment, at least in the progress of such cases,* we shall state shortly our reasons for

* To enumerate the dangers likely to result from assuming an identity of diseases which have many relations with one another, and yet totally differ in their nature, would seem to be quite superfluous. Perhaps it may be mentioned as not among the least, the great risk, as in cases of painful tumor of the mammæ, of subjecting patients unnecessarily to distressing operations. A case of difficult respiration occurred in the Royal Infirmary of Edinburgh, some months since, apparently spasmodic, in which a surgeon of eminence resorted to the operation of tracheotomy. It was that of Elizabeth Rattray, aged thirty-two, reported in the Medical Gazette for August 22, 1829. If we may venture to judge from a short statement, we should say this was a pure case of spinal irritation, and that the palpitations, hoarseness, and difficult breathing were referrible to disordered function of the cord, from its superior part, including the origin of the pneumo-gastric nerves, to the sixth or seventh dorsal vertebra. Had an examination been made, tenderness would inferring the existence of spasmodic croup as a distinct complaint, and offer a new reply to some queries on the subject, which Dr. Cheyne proposes to his friend, Dr. Kellie.

1. From analogy with the diseases of other organs, we may infer that affections of the nerves supplying the larynx and trachea must closely resemble organic affections of the same parts, and we necessarily suppose the nerves are sometimes the subject of disorder.

2. We can, by an effort of the will, imitate almost all the respiratory phenomena of croup.

3. We find certain cases of croup intermit suddenly, often leaving the patient perfectly free from complaint until the recurrence of the fit; a phenomenon wholly irreconcilable with the known character of inflammation.

4. We find intense cases of intermittent croup occurring as symptoms in diseases which we know are dependent upon an irritable or morbid state of the nervous system.

5. We know no distinctions of violent or mild between inflammatory and spasmodic croup, as regards symptoms: they are usually, at their onset, equally distressing, and sometimes the spasmodic is the more alarming or violent of the two. The result bears no relation to the intensity of the symptoms, and must therefore have reference altogether to some difference in the nature of the attack.

6. One is often relieved by antispasmodic remedies, the other scarcely ever; one seldom requires bloodletting, the other almost always; one is sometimes spontaneously cured, the other never.

The following are the questions proposed by Dr. Cheyne to Dr. Kellie.

have been found all along this track, the cure of which might perhaps have relieved all the distressing symptoms. That there was no chronic disease going on in the larynx or trachea, is evident from the absence of painful cough and fever, and, above all, from the relief obtained by the artificial opening, and the rapidity with which the wound was permitted to heal up. No organic disease, which had been coming on for three months, could have been benefited by the operation, or improved so much in a few days as to make it advisable to remove the tube. On the other hand, if it was looked upon as altogether a spasmodic attack, what was the value of an operation capable of affording relief only while the wound was kept open, and which, so long as the original cause remained, gave no security against a return of the oppression? We venture to assert, if the state of this patient since she left the hospital be inquired after, it will be found she has had a recurrence of the disorder, unless she has since been under medical treatment. It cannot be contended the danger of suffocation was so imminent, that, in any view, tracheotomy was requisite, as spasmodic affections of the larynx must be regarded in the same light with a somewhat similar one of the bronchial tubes in the asthmatic paroxysm, in which, however terrific the difficulty of respiration, absolute suffocation is scarcely ever known to occur.

1. Does this (Case I. in Dr. Cheyne's treatise,) appear to you to have been a case of spasmodic croup?

It seems merely necessary to answer in the affirmative, as, from observations already made, the distinctions between it and the inflammatory must be sufficiently obvious.

2. Have you ever known spasmodic croup brought on by cold?

Most frequently. In fact, diseases dependent upon disturbance or irritation of the nervous system are as readily brought on by cold as by any other cause. Spasmodic croup is in this respect precisely like spasmodic or nervous asthma, fits of which we have sometimes seen excited by cold, sometimes by disordered stomach, fatigue, or dissipation. We cannot tell why the nerves supplying the larynx or trachea should be simply disturbed in these cases by cold, any more than we can why the supra-orbitar or facial branches of the fifth become affected with pain from the same cause, when inflammation would appear to be its most natural result in both.

3. Have you known the same child, at different times, affected with spasmodic and inflammatory or genuine croup?

Though we cannot immediately call to mind any such instances, we entertain no doubt of its occasional occurrence. Affections of nerves, when obstinate, sometimes lead to inflammation. Spasm of the stomach, if unrelieved, may end in gastritis; colic in enteritis; hysteralgia in inflammation of the uterus. It must be remarked, however, that habits peculiarly disposed to spasmodic attacks, as the hysteric and nervous, seem much less liable to genuine inflammation than other constitutions. This is so generally true, that even when the appearances of local inflammation are present in hysterical or nervous persons, there is strong ground for suspecting the attack is (to adopt the usual phrase) only simulated.* Although admitting the occasional supervention of inflammatory croup with patients labouring under the spasmodic, analogy would therefore lead us to infer that it is not a very usual occurrence, and that a child who had once been attacked with spasmodic croup was much less liable to an attack of inflammatory, we will not say than if he never had any disorder of the air-passages, but than if he had once before been affected with the last-named disease. In our own experience, we have

* Dr. MARSHALL HALL makes many interesting remarks upon this important subject. "On some Diseases of Females," ch. vi.—EDITOR.

found children affected with spasmodic croup, very liable to a recurrence of it; much more so than those once attacked by the inflammatory are to a return of the inflammation. As it has been said the inflammation of croup, and its result, the production of a false membrane, are peculiar, and totally unlike common inflammation of any other or even of the same parts, it may perhaps be a question whether, admitting that spasmodic croup sometimes ran into the inflammatory, this is the species of inflammation that would be induced, or whether it might not rather be common laryngeal, or tracheal, or bronchial inflammation?

4. Have you ever observed spasmodic and genuine croup in the same family?

5. Do you recollect to have seen any case which you considered as spasmodic croup, pass into the genuine?

6. Did you ever, in spasmodic croup, observe difficult breathing continue after the immediate effect of the fit of croupy coughing was over?

The observations above may serve equally well as replies to the fourth and fifth questions. To the sixth we may offer Dr. Kellie's answer, that we have seen a degree of dyspnœa and short wheezing respiration in the interval of the fits.

7. Were the difficult breathing to continue, do you think it would be safe, in the cure, to trust to calomel alone?

If the difficult respiration continued, accompanied by febrile heat, we should say it was unsafe to trust to any one remedy. There is in many diseases the greatest difficulty in ascertaining whether an attack is dependent on inflammation or on nervous disturbance; and this difficulty is yet greater in croup, as it is chiefly a disease of children, in whom it is not easy to ascertain the existence of spinal tenderness. It is therefore incumbent on us, when any doubt is felt, to treat the case as inflammatory; and fortunately, in the early stages, the same remedies apply almost equally well to both. It is chiefly in the progress of spasmodic croup that mischief may be done by bleeding and depletion, and it will always sufficiently declare itself before these can be carried to a hazardous extent. We cannot agree with Dr. Mason Good, that, in the spurious attack, bloodletting, by increasing the nervous irritability, is always injurious: it does so only when employed largely or repeatediy; and, on the contrary, when cautiously done and in small quantity, we have known it allay the irritability of the system more speedily than other remedies.

8. Did you ever attend a case of spasmodic croup which terminated fatally?

Though we do not recollect ever to have seen a fatal case of spasmodic or spurious croup, we are quite convinced the disease sometimes terminates fatally. When spasm of the sphincter can prevent the flow of water from the bladder for hours, sometimes until it bursts, need we wonder that spasm of the glottis should occasionally persist until the patient is suffocated. Were examinations universally made in fatal cases of croup, we venture to predict we should soon have evidence of this termination, in the absence of the false membrane.

We have only to remark in conclusion, that there appears to be no resemblance between spasmodic croup and asthma, which could admit of their being mistaken for a moment. However croupy the respiration in asthma, and we have sometimes heard it intensely so, the patient himself is always aware, and, if old enough, will tell, that the difficulty is in the chest, and not in the throat. We must also agree with Dr. Kellie in the opinion that there never exists any inflammatory action of the pulmonary organs in spasmodic croup, at least at its outset; the febrile action accompanying its accession is almost invariably dependent on nervous irritation.

We shall now speak of an affection usually attacking infants, which may be considered as a species of the spasmodic croup.

This attack is seldom attended by wheezing or croupy respiration, as if it was simply an affection of certain motory nerves constricting the larynx. Dr. Hamilton, jun. of Edinburgh, treats of it among convulsive disorders of children, and describes it as a convulsive stricture of the upper part of the windpipe, characterized by a peculiar crowing sound, quite momentary, generally happening on the child's awakening from sleep, or on taking food, or when teazed or irritated. Dr. J. Clarke describes it more minutely, as characterized "by distinct attempts to fill the chest, between each of which a squeaking noise is often made; the eyes stare, and the child is evidently in great distress; the face and extremities, if the paroxysm continues long, become purple; the head is thrown backward, and the spine often bent as in opisthotonos; at length a strong expiration takes place, a fit of crying generally succeeds, and the child, evidently much exhausted, often falls asleep. Sometimes, but not frequently, in one of these attacks the infant dies."

The treatment recommended by Dr. Hamilton consists in attention to the state of the child's gums, keeping the bowels free, and applying stimulating liniments or blisters to the throat and chest; but viewing the respiratory affection, whatever its remote cause may be, as directly dependent on irritation at the origin of the eighth pair of nerves, it would seem that more advantage might be derived by directing our remedies to this point. Leeches and counter-irritation at the neck, followed by antispasmodics, or, if there was no febrile heat, by minute doses of some of the metallic oxyds, would, we have little doubt, in most instances interrupt the attack. In short, there appears to be no reason to treat it differently from similar affections in the adult depending on spinal irritation, but that in childhood, in which an extreme delicacy of organization prevails, it should be constantly held in mind that the diseases of irritation are always more serious, and more apt to be attended with danger, than in advanced life.

Dr. J. Clarke believes that this, and indeed all other convulsive affections of children, depend upon some organic affection of the brain. He details the post-mortem appearances in a few cases in illustration, and says that "all the arguments founded on the doctrine of sympathy and irritability are drawn *ab ignoto*, and it seems much more conformable to reason and observation to infer that such convulsive affections arise from some derangement of organization, however temporary, than to resort for an explanation of them to imaginary causes, and such as offer to the mind no satisfactory conclusions."

In reply to this reasoning, it may be remarked that our knowledge both of the physiology of the brain and spinal marrow, and the pathology of its many diseases, is far too obscure to allow of our drawing any inferences not warranted by established facts. It is surely more philosophical to infer change of structure only where we find it, and to suppose some other state capable of disordering the functions of parts, where we do not find it, especially when such conclusions seem strikingly confirmed by a fact that might almost suggest itself: the slowness, the imperfection, or impossibility of cure in the former; the suddenness, and perfection, and facility with which it is often accomplished in the latter. No one is so ridiculous as to suppose that *no change* takes place in functional disorder, but it would certainly seem that in such as are said to depend on irritation *no change of structure* takes place, no deranged organization: a person ascending in a balloon at a certain height becomes oppressed in consequence of the rarity of the air, not from any change or breach in the mechanism of his frame, but because of the altered relation between that frame and the atmosphere.

We have felt it necessary to dwell a little on this subject, from a conviction of the great importance, in all disorders of the system, of distinguishing the organic from the functional. We are quite of Dr. Underwood's opinion, that, even when convulsive affections prove suddenly fatal, they are most commonly sympathetic, or dependent on irritation; and in believing this, we are treasuring up for ourselves new hopes for our remedies, and increased zeal in their application.

From all that has been said, it will not appear remarkable that irritation affecting the upper portion of the spinal column should sometimes exhibit symptoms closely resembling those of hydrophobia. In fact, hydrophobia itself, as Hufeland and Dr. Reid, of Dublin, believed, would seem to be a disease, though of a peculiar nature, whose seat is entirely in the spinal marrow, and which seems to affect the superior part, and particularly that portion of it which is allotted to the function of respiration. The manner in which it commences, and the symptoms which it gives rise to in its progress, as well as the mode of its termination and the appearances on dissection, all confirm this opinion. The pain commencing in the wound or cicatrix, and proceeding from thence to the back of the neck, and, when the wound is in the hand or arm, following, or rather falling into, the course of the spinal, accessory, or external respiratory nerve of Mr. Bell. The freedom of the intellectual functions from disorder in the commencement, the derangement of the digestive organs, loss of appetite, nausea, vomiting, constipation, and sometimes colic; the pain in the back of the neck; the difficulty of swallowing liquids, and violent spasm of the glottis, and convulsive affection of all the respiratory muscles whenever the attempt is made, and the fatal event being always the result of a failure of the action of respiration, are all symptoms which point out a derangement of the functions of nerves which have their origin at the upper portion of the spine; the exquisite acuteness of all the senses, the excessive sensibility to light and sound, and other impressions, is quite in accordance with this idea; since, if the disease commences in the upper part of the spinal marrow, it is impossible to suppose that the fifth nerve, which is so necessary to the functions of the senses, could, from the nearness of its origin, escape being implicated. As it is a disorder that always terminates fatally, it is not at all surprising that, in the progress of it, the whole nervous system should become affected, and excessive nervous excitement, violent general convulsions, and high delirium, should, before its close, be added to the symptoms above mentioned. The appearances on dissection confirm the fact of the spine being its seat; though, from the remark we have made above on the constant fatality of its termination, it would seem that morbid anatomy cannot, except after a great number of dissections, indicate precisely that part of the nervous system where it commences; since, if the entire of this system is violently affected before its conclusion, the entire, from the intimate connexion of the whole, would probably exhibit morbid appearances. Thus it is observed that traces of inflammation are found in the brain or its membranes, in the cerebellum or its membranes, and in the membranes of the medulla spinalis. However, what is more to our purpose is, that inflammatory appearances are never found wanting in the bronchial tubes, scarcely ever in the trachea about its bifurcation, and seldom in the larynx. From all these circumstances, perhaps, it may not be too much to hope that something may yet be done in the prevention of this dreadful disorder, by early attention to the spine. Instances bearing a striking similitude to it, and occurring as symptoms of cervical irritation, will appear in subsequent papers.

We shall conclude the observations on disorder of the respiratory functions with an interesting case of spinal irritation, in which the transference of the diseased action from one set of organs to another, precisely as it shifted its seat in the cord, was remarkably obvious.

XXXII. A lady, aged forty-five, was attacked with violent headach, affecting especially the brow and forehead, and attended by violent throbbing of the temporal arteries, with flushed face and feverishness. The pain of head was dreadful at times, and usually much worse at the right than at the left side. She has been subject to it for years, though seldom to such a degree as at present, and generally gets some relief by going to bed. The attack comes on in rather a singular manner: her vision first seems suddenly dim or troubled, or the half of any object she looks at disappears. If her eyes are fixed on the window, the glass appears to move like flowing water in the sunshine; or, if engaged in reading, one half of the letters seem wanting. This is an infallible precursor of the pain, and, as soon as it occurs, she gives up whatever employment engages her at the moment, and prepares to meet it. Her stomach soon becomes sick, and this is followed by the violent pain in the head, which continues an uncertain period, from a few hours to three or four days. Her right eye is usually affected first, but eventually both. Her general health appears good; her habit inclines to fulness. She was very ill some years since, with constant palpitation and nervous catchings or startings when in bed; oppression; pain in the chest, arms, neck, and stomach: organic disease of the heart was suspected at the time by her medical attendant; but another physician, who was consulted, treated it entirely as an hysterical affection, and she recovered under the use of mild aperients and nervines. As there was some fulness of pulse, with feverishness and excessive action of the temporal arteries, leeches were in this instance applied to the temples, which, with active purgatives, removed the attack. No examination of the cervical portion of the spine was made.

About twelve months after, we were called up in the night to this lady, who said she was dying. It appeared that, some hours after retiring to rest, she was suddenly awakened by violent palpitation, with a shooting pain in the region of the heart, extending from thence down the shoulder and arm; she started out of her sleep, pale and terrified, and felt as if she was about to die, but in a short time the pain lessened, and the palpitation began to subside. On arriving at the house, she appeared much relieved, but a feeling of languor and apprehension remained. As our attention was at this time beginning to be directed to functional affections of the cord, the cervical vertebræ were examined, but no tenderness was detected: she was treated with aperients, camphor, and other antispasmodics, but the palpitation continued to recur for several nights, though in a much less degree. As it subsided, she began to complain greatly of the back of the neck; the muscles were so sore that she could not bear the gentlest friction, nor could she turn her head or stoop without pain; there was also pain round the throat, and down either shoulder; she had uneasiness in the neck when this attack first came on, but her terror at the palpitation prevented her attending to it. She said, when examined, that the pressure was not made low enough. There was now excessive tenderness of the lowest cervical vertebra.

In the following summer, we were again summoned at midnight to visit this lady. She was awakened out of her sleep, as on a former occasion, with a sudden fit of violent oppression, in which she thought she would have died; her breathing was shrill and stridulous, threatening imminent suffocation, exactly as in the worst cases of spasmodic croup. The paroxysm was over on our arrival, but a croupy wheezing respiration remained, and she was in much apprehension of a return of the attack. There was extreme tenderness of the first, sixth, and seventh cervical vertebræ. She speedily recovered from this distressing affection; but, after another interval of some months, was seized with pains in the hip, knee, and ankle, which came on in paroxysms of intense severity, darting down the limbs, and attacking the joints severely; there was superficial soreness of the muscles, but no increased heat, and little general feverishness. This last disorder (a true neuralgia) was readily relieved; but as, on her recovery, the patient is always unwilling to persevere in attention to the spine, or follow up any regular plan of prevention, there is no doubt but she will hereafter become the subject of some new symptoms; perhaps of comatose fits, or syncope, or a recurrence of any of the old attacks.

[To be continued.] Vol. lxiv. p. 397.

---

CONVULSION.

